# MultiDataSet: an R package for encapsulating multiple data sets with application to omic data integration

**DOI:** 10.1186/s12859-016-1455-1

**Published:** 2017-01-17

**Authors:** Carles Hernandez-Ferrer, Carlos Ruiz-Arenas, Alba Beltran-Gomila, Juan R. González

**Affiliations:** 1 Institut de Salut Global de Barcelona (ISGlobal) - Campus Mar, Barcelona Biulding: Biomedical Research Park, c/Dr. Aiguader, 88, 08003 Barcelona, Spain; 2 Universitat Pompeu Fabra (UPF), Barcelona, Spain; 3 CIBER Epidemiología y Salud Pública (CIBERESP), Barcelona, Spain

**Keywords:** Omics data, Data integration, Data infrastructure, Data organization, R

## Abstract

**Background:**

Reduction in the cost of genomic assays has generated large amounts of biomedical-related data. As a result, current studies perform multiple experiments in the same subjects. While Bioconductor’s methods and classes implemented in different packages manage individual experiments, there is not a standard class to properly manage different omic datasets from the same subjects. In addition, most R/Bioconductor packages that have been designed to integrate and visualize biological data often use basic data structures with no clear general methods, such as subsetting or selecting samples.

**Results:**

To cover this need, we have developed MultiDataSet, a new R class based on Bioconductor standards, designed to encapsulate multiple data sets. MultiDataSet deals with the usual difficulties of managing multiple and non-complete data sets while offering a simple and general way of subsetting features and selecting samples. We illustrate the use of MultiDataSet in three common situations: 1) performing integration analysis with third party packages; 2) creating new methods and functions for omic data integration; 3) encapsulating new unimplemented data from any biological experiment.

**Conclusions:**

MultiDataSet is a suitable class for data integration under R and Bioconductor framework.

**Electronic supplementary material:**

The online version of this article (doi:10.1186/s12859-016-1455-1) contains supplementary material, which is available to authorized users.

## Background

Projects in biotechnology are constantly generating new types of data. As the reduction of costs of genomic assays drops, volume as well as data diversity greatly increase. While advances in knowledge of personalized medicine are expected, scientists are continuously challenged on data management and analysis [1, 2].

Bioconductor started in 2001 as an initiative to provide free software, written in R and implemented in standardized methods and classes, to analyze high-throughput biological data [3, 4]. Currently, Bioconductor is a public repository where scientists can find R packages to analyze all kinds of high-throughput omic data (e.g. genomic, transcriptomic, epigenomic, proteomic…). A standard infrastructure has been created to represent biological data comprising, amongst others, two basic R classes to load experiment’s information: *eSet* and *SummarizedExperiment*. The main objective of these classes is that biological data and phenotypic descriptions are well coordinated. In particular, methods such as subsetting are easily applied simultaneously to experiment and phenotypic data. While *eSet* is designed for microarray data, *SummarizedExperiment* is for next generation sequencing data.

Major public projects have performed experiments to a group of individuals generating different types of datasets [5]. For instance, the Cancer Genome Atlas (TCGA) [6, 7], is the largest resource available for multi-assay cancer genomics data; the 1000 Genome Project [8, 9] aims to provide a comprehensive resource for human genetic variants and gene-expression across populations and; the International Cancer Genome Consortium (ICGC) [10, 11] coordinates 55 research projects to characterize the genome, transcriptome and epigenome of multiple tumors. In addition, large repositories collect data of several smaller projects allowing unified storage and stimulating data sharing. Gene Expression Omnibus (GEO) [12–14] is the primary database where data from multi-assay experiments is shared publicly. Other reference databases are dbSNP [15, 16], a deposit for short genetic variations and Database of Genomic Variants archive (DGVa), for longer structural variants [17, 18].

All of these data resources are accessible through standard Bioconductor classes (*eSet* and *SummarizedExperiment*). There are numerous packages used to retrieve and transform data from public repositories. For instance, GEOquery [19] that obtains GEO data as an *eSet* object. Such packages aim to facilitate downstream analyses for Bioconductor’s packages. However, Bioconductor lacks a standard class to efficiently manage different datasets obtained from the same individuals.

Several R/Bioconductor packages have implemented methods to integrate and visualize biological data: *PMA* [20–22], *mixOmics* [23–25], *made4* [26, 27], *RGCCA* [28, 29], *omicade4* [30, 31], *CNAmet* [32, 33], *RTopper* [34, 35], *iClusterPlus* [36, 37] and *STATegRa* [38] among others. Each of these packages implements a different strategy to face the integration analysis. They typically use their own data structure, which is usually a list of matrices. The use of such structure makes it difficult to perform usual operations such as subsetting data across data sets and selecting samples (e.g. complete cases are usually required in all integration analysis). The specificity of the data structures to each method further hinders the user’s disposition to perform different integration analyses on one study. Therefore, a standard structure to manage the different datasets of the same individuals will promote the use of current and future integration methods, allowing the implementation of general methods for management and processing.

In this article, we present *MultiDataSet*, a new R class based on Bioconductor standards developed to encapsulate multiple datasets. *MultiDataSet* deals with the usual difficulties of managing multiple and non-complete datasets while offering a simple way of subsetting features and selecting individuals. We describe the internal structure of *MultiDataSet* and illustrate its use in three examples (Additional files 1, 2 and 3) which cover three common situations in integration analyses.

## Design and implementation

*MultiDataSet* is a S4 class of R implemented under Bioconductor guidelines [39]. Its structure is an extension of the abstract *eSet* class. *MultiDataSet* is therefore a data-storage class that comprises datasets of different omic data (assay data), feature data and phenotypic data. Despite its general form, *MultiDataSet* maintains the specific characteristics of the datasets (e.g. it preserves matrices of calls and probabilities of a *SnpSet*).

### Internal structure of MultiDataSet

**Fig. 1:**
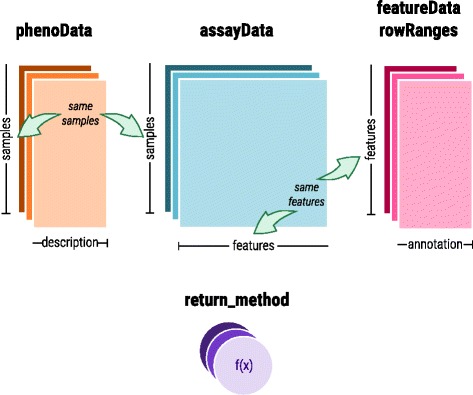
This schema shows how the information is stored in the five attributes of a MultiDataSet and how the different parts are linked. phenoData and assayData share the dimension corresponding to samples. featureData, rowRanges and assayData share the dimension corresponding to features. All the attributes are linked through the data-sets name

*MultiDataSet* comprises five fields that are R standard lists. Their names match other Bioconductor classes: *assayData* that contains the measurement values; *phenoData* that stores the description of the samples; *featureData* and *rowRanges* that have the description of the features; and *return_method* that allows recovering the original dataset. Relation between fields is shown in Fig. 1. In each dataset, samples are shared between *assayData* and *phenoData*, and features between *assayData*, *featureData* and *rowRanges*. We have programmed a function to recover the original datasets. The class is designed such that the different data is coordinated. A particular feature of *MultiDataSet* is the storing of datasets from different experiments that may not share the full set of samples between them.

Six accesors are available to retrieve information from each *MultiDataSet*’s fields: *assayData*, *pData*, *fData*, *rowRanges*, *rowRangesElements* and *sampleNames*. The first four retrieve the content of *assayData* (a list of environments), *phenoData* (a list of *AnnotatedDataFrames*), *featureData* (a list of *AnnotatedDataFrames*) and *rowRanges* (a list of *GenomicRanges* with NAs for the datasets with features without genomic coordinates). *rowRangesElements* returns the names of datasets with a genomic coordinates in a *GenomicRanges*. The accessor *sampleNames* returns a named list with the samples names of each data set.

### Adding datasets to MultiDataSet

Following Bioconductor guidelines, *MultiDataSet* objects are created empty through its constructor. Once the object is created, datasets can be added with *add_eset* and *add_rse*. The first function adds an object of class *eSet* while the second adds a *SummarizedExperiment* object and its extensions. The two functions have the same arguments: the *MultiDataSet* object, the dataset to be added, a tag for the type of dataset (i.e. methylation, expression…) and a name for each dataset. *MultiDataSet* thus allows the storage of multiple dataset of the same type, under different names. For features with genomic coordinates, a *GenomicRanges* object is created from the dataset’s *featureData*. In order to maintain the consistency across all datasets, the names of the samples are given by those in the phenotype dataset (a column called “*id*” is requested). If not present, object’s *sampleNames* are used.

*MultiDataSet* package incorporates three specific functions to include specific omic data sets: *ExpressionSet* (*Biobase* package), *MethylationSet* (*MultiDataSet* package) and *SnpSet* (*Biobase* package). These specific functions call general functions to add the data after performing extensive or specific checks (e.g. checking the class of the set or checking *fData’*s columns). As a result, only datasets with defined features can be introduced to *MultiDataSet* through a specific function.

**Fig. 2:**
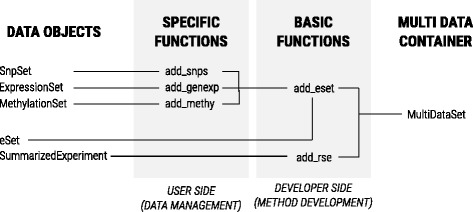
This figure represents the organization of the specific and basic functions to add datasets to a *MultiDataSet *object. The basic functions, that receive generic *eSet *and *SummarizedExperiment *objects, directly interact with the *MultiDataSet *objects and developers should use them to extend the functionality of *MultiDataSet*. Specific functions receive more specific datasets and interact with the *MultiDataSet *object through basic functions. They check the structure of the dataset and users should use them

Specific functions should always be used by users to ensure that the sets are properly added to *MultiDataSet*. The two basic functions *add_eset* and *add_rse* are intended to be used only by developers to develop new specific functions. The hierarchy between the specific and basic functions is shown in Fig. 2.

### Subsetting MultiDataSet

We have implemented two methods to perform subsetting. The operator ‘*[*’ can be used to select individuals, datasets and/or features. In the case of having tables with different samples, subsetting is performed by considering the union of samples from the different tables. For instance, let us assume that table 1 contains individuals A and B. Table 2 has individuals A, B and C, and Table 3 is having individuals A, C. Let us also assume that we are interested in getting information from tables 1, 2 and 3 for individuals A and C. Our subsetting method will return a *MultiDataSet* object containing individuals A for table 1 and individuals A and C for tables 2 and 3. We think that this procedure is better than returning a *MultiDataSet* object only having individual A (i.e. intersection) for the three tables. Therefore, subsetting by individuals may not return complete cases. Notice that the package has another function (*commonSamples*) that can be applied to this object to get complete cases if necessary.

When subsetting by datasets, if only one is selected, the original dataset is returned (aka. *SnpSet*, *MethylationSet*…). *GenomicRanges* object can be used as an argument to select the features present in a given genomic range. In this process, sets with no genomic coordinates (e.g. metabolomic data) are discarded. We extended the subsetting function of R (*subset*) to select specific features within a dataset, such as features associated to a gene or filtering individuals given a phenotype.

## Results

We illustrate three common situations where *MultiDataSet* is useful. The examples are used to show package strengths and advantages. The first example shows how to use *MultiDataSet* to perform two integration analyses with already existing tools (R packages *omicade4* and *iClusterPlus*). Second example shows how to develop a function to integrate gene expression and methylation data. Third example illustrates how to incorporate a new type of dataset that is not available in *MultiDataSet* to store proteome data including limit of detection.

### Using MultiDataSet with third party R packages

In the first example, we show how to perform an integration analysis with three datasets using two approaches: 1) multivariate co-inertia analysis and 2) clustering of multiples tables. These methods are implemented in the R packages *omicade4 *and *iClusterPlus *(see code in Additional file 1). For both analyses, we integrate three datasets stored in a single *MultiDataSet *object. We use two gene expression datasets and one methylation dataset. The analysis includes three steps. First, the different datasets are added to a *MultiDataSet *through specific functions. Second, a *MultiDataSet *method is used to easily select those samples present in all the datasets. Finally, a wrapper is used to pass the *MultiDataSet *object to mcia function (from *omicade4*). This process is also done for the clustering analysis of *iClusterPlus*. Both wrappers, for *mcia *and *iClusterPlus*, are included in *MultiDataSet *package. We also provide the code of the wrappers to encourage developers to create their own.

### Integration of methylation and expression using MultiDataSet

In the second example, we show how to develop a function to implement a new method to integrate two types of data. We describe the different parts of *correlationMethExprs*, a function from *MEAL* package [40] that performs an integrative analysis of methylation and expression data. The function has four main steps: 1) checking of data, 2) preparation of data structures, 3) implementation of the integration algorithm and 4) formatting of the results. Checking the correct format of original datasets is performed whenever the sets are added to the *MultiDataSet*, so an integration function only needs to check the global structure of the *MultiDataSet. MultiDataSet*’s methods facilitate the preparation of data structures and the recovery of original data in Bioconductor classes for the use of any Bioconductor’s methods. In addition, we illustrate common steps of an integration analysis that are easily performed thanks to commonSamples joint to the list conversion (*as.list*).

### Adding a new type of data to MultiDataSet objects

In the third example, we show how to create a new specific function to deal with a type of data that is not natively supported in *MultiDataSet *package (see Additional file 3). We also cover how to implement checks of the object’s structure inside the function to ensure *MultiDataSet*’s consistency. This is illustrated by extending an *eSet *object to include proteins’ data. Detectors used in multiplex protein quantification work inside the dynamic range of protein concentrations. However, real protein concentrations may be outside these ranges so values outside the limit of detection (LOD) are generated. To address this issue, we introduce a new function to load protein data and to modify values out of the LOD. Then, we implement *ProteomeSet*, an *eSet *extension, to include the original data, the transformed data and the description of the limit of detection of each protein. Finally, we develop a new specific function to add *ProteomeSets *and to check that it contains all the components required with the right names. *MultiDataSet *can thus be extended to potentially contain any data type.

## Discussion

The reduction of costs of microarray technologies and next generation sequencing has promoted the expansion of projects that collect different type of biological data on individuals. Consequently, it is useful to have a standard procedure to combine the information from different sources for further processing and analysis. We have developed *MultiDataSet* as a package that helps to storage different datasets, including common Bioconductor’s classes. *MultiDataSet* can also be extended to store novel data types.

*MultiDataSet* is implemented so it runs extensive checks on the datasets being integrated. It checks for the consistency within and across datasets. In addition, regular operations like selecting complete cases or subsetting are included in the package. *MultiDataSet* design facilitates data validation, as each kind of omic data is introduced using a specific function. These specific functions include the dataset’s type in the objects’ name, thereby standardizing *MultiDataSet* objects. Integration functions thus require fewer checks because many are done in the previous steps (see Additional files 1, 2 and 3). In addition, the checking can be modified by re-implementing a specific function, without altering the structure of the package.

*MultiDataSet* is designed to manage multiple datasets while preserving their original characteristics. Thus, the assay data, the phenotypic data and the feature data are stored independently for each dataset. In the case of the phenotypic data, we prefer this approach by several reasons. In omic datasets, the phenotypic data also stores technical information. For instance, if we were interested in encapsulating two *MethylationSets*, we will have technical variables with the same name but different information. Storing this information in one pheno data will lead to rename both columns to keep the information. In studies with longitudinal data, some phenotypic variables of the individuals change in the datasets. As an example, if we had two ExpressionSets, one obtained at age 4 and the other at age 18, the variable age will have very different values in each dataset. Finally, we decided to keep the pheno data separately to allow the user recovering the original datasets. Consequently, users can create their *MultiDataSet* with different datasets, perform some subsetting operations (e.g. selecting complete cases) and then recover the objects with the original classes that can be used to perform downstream analysis.

*MultiDataSet* performs common subsetting operations as well as managing experiments with multiple omic sources. It contains functions to select the individuals with information in all datasets or those features of each dataset that are inside a region of the genome. Moreover, *MultiDataSet* can also perform other complex subsetting operations, such as selecting subjects with specific phenotypes or selecting features belonging to a gene, which can be of great help in candidate gene studies. In addition, *MultiDataSet* easily recovers the original dataset for the use with native Bioconductor functions.

A Bioconductor group interested on combinable experiments has started a repository (https://github.com/vjcitn/MultiAssayExperiment). This group aims to use R/Bioconductor to define interfaces allowing efficient selection and combination of high-dimensional assays. However, it seems that the project focuses on TCGA. The package, called *MultiAssayExperiment*, shares similar capabilities for managing data, specially related to subsetting. Nevertheless, addition of sets is not standardized, lacking some of the advantages that we mentioned in the previous sections. Overall, we think that *MultiDataSet* is a strong candidate to become a standard class in integration of multiple omic data sets since it is already available in Bioconductor (https://bioconductor.org/packages/release/bioc/html/MultiDataSet.html), while *MultiAssayExperiment* is still under development. Another disadvantage is that *MultiAssayExperiment* is being developed by a large number of contributors making package improvements slow and tedious.

One of the limitations of *MultiDataSet* is that the memory management is not optimized as its uses straightforward extensions of Bioconductor’s classes. As datasets grow in size, further developments on the storage need to be devised. We have used *MultiDataSet *to encapsulate two *MethylationSets *having 450 K CpGs, on SnpSet with 800,000 SNPS and an *ExpressionSet *having 70,000 probes without problems. The other limitation is that, nowadays, *MultiDataSet* can only be used with three integration pipelines: by *mcia* (*omicade4*) and *iClusterPlus* through a wrapper and as input object for *MEAL* package.

Currently, we have two lines of work to increase the usage of our package. On one hand, we are contacting integration methods developers to allow using *MultiDataSet* objects as input in their pipelines. On the other hand, we are developing a new function to download data from GEO and include them in a *MultiDataSet* object.

## Conclusion

*MultiDataSet* is a new R class designed to manage data from different omics experiment in the same individuals. It includes methods to perform typical operations, such as subsetting or selecting common samples, making integrative analysis more accessible to users with a low expertise in bioinformatics. In addition, its implementation eases standardization of multiple omic sets, which will also ease the development of new integration functions. Altogether, *MultiDataSet* is a suitable standard for data-storage in integration analysis done under R and Bioconductor framework.

## Availability and requirements

Project name: MultiDataSet

Project home page: https://bioconductor.org/packages/release/bioc/html/MultiDataSet.html

Operating Systems: Windows, UNIX and MAC;

Programming Language: R

Other requirements: none

License: MIT.

## Additional files

Additional file 1: “Using MultiDataSet with third party R packages”. This file illustrates how to perform an integration analysis using *multivariate co-inertia analysis* (omicade4) and *clustering of multiples tables* (iClusterPlus). (ZIP 38 kb)
Additional file 2: “Integration of methylation and expression using MultiDataSet”. This file is a tutorial of how to develop a function to implement a new method using MultiDataSet capabilities. (HTML 78 kb)
Additional file 3: “Adding a new type of data to MultiDataSet objects”. This file exemplifies how to create a method to incorporate data from S4 classes to a MultiDataSet object. (HTML 66 kb)

## Abbreviations

DGVs: Database of genomic variants archive
GEO: Gene expression omnibus
ICGC: The International Cancer Genome Consortium
LOD: Limit of detection
SNP: Single nucleotide polymorphism
TCGA: The cancer genome atlas
