# Supplementary material for: MultiDataSet: an R package for encapsulating multiple data sets with application to omic data integration
Source: BMC Bioinformatics. 2017 Jan 17;18:36. doi: 10.1186/s12859-016-1455-1 (PMC5240259; doi:10.1186/s12859-016-1455-1)
Supplement: Additional file 1: — “Using MultiDataSet with third party R packages”. This file illustrates how to perform an integration analysis using multivariate co-inertia analysis (omicade4) and clustering of multiples tables (iClusterPlus). (ZIP 38 kb) [file 12859_2016_1455_MOESM1_ESM.zip › sm1_MultiDataSet_UseCase_3party.html]

Using MultiDataSet with third party R packages


# Using MultiDataSet with third party R packages

#### *Carles Hernandez-Ferrer, Carlos Ruiz-Arenas, Alba Beltran-Gomila, Juan R. Gonzalez*

#### *2016-10-04*

#### Package version: MultiDataSet 1.1.6

# Contents

- 1 Introduction
- 2 Objective
- 3 Implementation
  - 3.1 Loading Data
  - 3.2 Packaging Data
  - 3.3 Integrative Analysis
    - 3.3.1 Interacting with `omicade4`
    - 3.3.2 Interacting with `iClusterPlus`
- 4 Summary and Conclusion
- 5 Appendix: Wrappers
  - 5.1 Wrapper for `omicade4`
  - 5.2 Wrapper for `iClusterPlus`

# 1 Introduction

Projects in biomedicine are generating information about multiple types of omics data. Most of these datasets are publicly available through on-line data backs like GEO (*Gene Expression Omnibus*), TCGA (*The Cancer Genome Atlas*) or EGA (*European Genome-phenome Archive*) among others. Standard association analyses between a given phenotype and a single omic dataset (aka. SNPs, gene expression, methylations,…) are insufficient to address most of the scientific questions that rely on knowing the mechanisms involved in complex diseases. Methods to properly integrate multi-data sets have to be used to analyze such amount of information.

One of the computational challenges when dealing with multi-omic data is to define an easy way to compact multiple pieces of information. This methodology must allow retrieving data for common-samples, subsetting individuals having a specific characteristic or selecting features from a given genomic region or a selected gene. `MultiDataSet` is conceived as a container and manager of multiple omic datasets while allowing performing common object operations such as accession to elements or subsetting among others. The vignette of `MultiDataSet` describes how to manipulate a MultiDataSet object as well as how to perform other basic operations.

In this document, we learn how to create a `MultiDataSet` object to be used in an integrative analysis implemented in `omicade4`. This Bioconductor package implements multiple coinertia analysis (mcia), a method that generalizes principal component analysis (PCA) in the case of having more than two tables. This package deals with multi-omic data by using lists. However, it requires analyzing complete cases (i.e. having common samples across datasets). Here, we illustrate how to encapsulate three different omic datasets (two microarray and one methylation experiments), how to get an object having complete cases and how to perform integration analysis. The same procedure is described in the case of using `iClusterPlus`, a method to perform cluster analysis having multiple datasets.

# 2 Objective

The objective of this document is to illustrate how to use `MultiDataSet` to store multiple omic datasets. We also illustrate how to use an object of class `MultiDataSet` in third party integration packages/pipelines. The document includes two different examples. One illustrates how to analyze a MultiDataSet object using `omicade4`, an R package designed to perform mcia analysis. The second example illustrates how to use `iClusterPlus` to perform clustering analysis on multiple datasets.

# 3 Implementation

This section includes three subsections. In the first subsection, **Loading Data**, three dummy datasets are loaded. These datasets correspond to two microarray experiments, which are encapsulated as an `ExpressionSet` and one methylation experiment stored as a `MethylationSet`. The second, **Packaging Data**, illustrates how `MultiDataSet` is used to package multiple pieces of information. Notice that our package allows the possibility of having datasets of the same type. The third section, **Integrative Analysis**, shows how to perform the integration analysis of the three datasets by using `omicade4` and `iClusterPlus`.

## 3.1 Loading Data

Two libraries are required to manage the three dummy datasets: `MultiDataSet` and `Biobase`. These libraries contain the required classes of objects we are dealing with: `MultiDataSet`, `ExpressionSet`, and `MethylationSet`:

```
library(MultiDataSet)
library(Biobase)
```

Three dummy datasets have been generated for creating this document. They can be downloaded from the Supplementary Material of the manuscript. The gene expression file (`expression.RData`) has two `ExpressionSets` corresponding to gene expression measured at two different time points. The methylation file (`methylation.RData`) contains a `MethylationSet`, which is having information about beta values of 450K CpGs. The three datasets belong to the same experiment.

```
load("expression.RData")
load("methylation.RData")
ls()
```

```
## [1] "gSet1" "gSet2" "mSet"
```

The result of the command `ls` from the previous chunk tells us that objects containing the information are called `gSet1`, `gSet2`, and `mSet`. The first two are the `ExpressionSets` while the last one corresponds to the `MethylationSet`. The following table shows the number of samples of each set and the number of common samples between each pair of sets:

|  | `gSet1` | `gSet2` | `mSet` |
| --- | --- | --- | --- |
| `gSet1` | 50 | 16 | 32 |
| `gSet2` | - | 27 | 19 |
| `mSet` | - | - | 41 |

The table should be read as:

- `gSet1` has 50 samples, `gSet2` has 27 samples and `mSet` has 41 samples
- `gSet1` and `gSet2` have 16 samples in common
- `gSet1` and `mSet` have 32 samples in common
- `gSet2` and `mSet` have 19 samples in common

The following three commands show the number of samples that each pair of objects has in common. They were used to create the previous table.

```
## gSet1 intersection gSet2
length(intersect(sampleNames(gSet1), sampleNames(gSet2)))
```

```
## [1] 16
```

```
## gSet1 intersection mSet
length(intersect(sampleNames(gSet1), sampleNames(mSet)))
```

```
## [1] 32
```

```
## gSet2 intersection mSet
length(intersect(sampleNames(gSet2), sampleNames(mSet)))
```

```
## [1] 19
```

The number of common samples along the three datasets is 12:

```
## gSet1 intersection gSet2 intersection mSet
length(intersect(intersect(
    sampleNames(gSet1), sampleNames(gSet2)), sampleNames(mSet)))
```

```
## [1] 12
```

## 3.2 Packaging Data

With the three sets into the current R session, we can proceed to package them in a `MultiDataSet` object. There are four required steps:

1. Create an empty `MultiDataSet` object
2. Add the content of the first `ExpressionSet`
3. Add the content of the second `ExpressionSet`
4. Add the content of the `MethylationSet`

An empty `MultiDataSet` is created by using the function `createMultiDataSet`. This empty object is called `md`:

```
md <- createMultiDataSet()
md
```

```
## Object of class 'MultiDataSet'
##  . assayData: 0 elements
##     . elements:  
##  . featureData:
##  . rowRanges:
##  . phenoData:
```

The generic function `show` of `MultiDataSet` objects informs that `md` contains zero elements (see `assayData` section). After that, the three sets can be added.

`MultiDataSet` has specific functions to add each type of dataset. The function `add_genexp` allows adding objects of class `ExpressionSet`. The following code adds `gSet1` to the empty `MultiDataSet` creating `md.e`.

```
md.e <- add_genexp(md, gSet1)
md.e
```

```
## Object of class 'MultiDataSet'
##  . assayData: 1 elements
##     . elements: expression 
##  . featureData:
##     . expression: 300 rows, 4 cols (chromosome, ..., end)
##  . rowRanges:
##     . expression: YES
##  . phenoData:
##     . expression: 50 samples, 3 cols (gender, ..., inv)
```

Now, `md.e` only contains a set of type *expression*. This set is *tagged* as `expression` and contains 300 features for 50 samples. Next step is to add `gSet2` to the `MultiDataSet`.

```
md.e <- add_genexp(md.e, gSet2)
```

```
## Error in add_eset(object, gexpSet, dataset.type = "expression", GRanges = range, : There is already an object in this slot. Set overwrite = TRUE to overwrite the previous set.
```

The function `add_genexp` raised an error. The error indicates that a set using the slot `"expression"` already exists.

`MultiDataSet` objects can only contain a single set with the same name. By default, `add_genexp` uses `"expression"` as the name of the new expression dataset. This name was used when adding the object `gSet1`, and therefore, it cannot be used again when adding `gSet2`. To solve this, `add_genexp` includes the argument `dataset.name` to specify the name of each dataset (`ExpressionSet`).

```
md <- add_genexp(md, gSet1, dataset.name = "gSet1")
md <- add_genexp(md, gSet2, dataset.name = "gSet2")
md
```

```
## Object of class 'MultiDataSet'
##  . assayData: 2 elements
##     . elements: expression+gSet1, expression+gSet2 
##  . featureData:
##     . expression+gSet1: 300 rows, 4 cols (chromosome, ..., end)
##     . expression+gSet2: 300 rows, 4 cols (chromosome, ..., end)
##  . rowRanges:
##     . expression+gSet1: YES
##     . expression+gSet2: YES
##  . phenoData:
##     . expression+gSet1: 50 samples, 3 cols (gender, ..., inv)
##     . expression+gSet2: 27 samples, 3 cols (gender, ..., inv)
```

The previous three lines of code depict how to add `gSet1` and `gSet2` to the empty `MultiDataSet` object called `md`. We observe that the resulting `MultiDataSet` object is containing two *expression* data. The `show` method also informs about the number of features and samples of each dataset. The names of each dataset added into the `MultiDataSet` object follows the following structure: the *type of dataset*, the character *+* and the *name of the dataset*.

Finally, the methylation data is added using the method `add_methy`:

```
md <- add_methy(md, mSet)
md
```

```
## Object of class 'MultiDataSet'
##  . assayData: 3 elements
##     . elements: expression+gSet1, ..., expression+gSet2 
##  . featureData:
##     . expression+gSet1: 300 rows, 4 cols (chromosome, ..., end)
##     . expression+gSet2: 300 rows, 4 cols (chromosome, ..., end)
##     . methylation: 300 rows, 17 cols (chromosome, ..., Regulatory_Feature_Group)
##  . rowRanges:
##     . expression+gSet1: YES
##     . expression+gSet2: YES
##     . methylation: YES
##  . phenoData:
##     . expression+gSet1: 50 samples, 3 cols (gender, ..., inv)
##     . expression+gSet2: 27 samples, 3 cols (gender, ..., inv)
##     . methylation: 41 samples, 4 cols (gender, ..., inv)
```

The final `MultiDataSet` object `md` encapsulates three datasets. This can be checked with the general functions `length` and `names`:

```
length(md)
```

```
## [1] 3
```

```
names(md)
```

```
## [1] "expression+gSet1" "expression+gSet2" "methylation"
```

## 3.3 Integrative Analysis

Once the `MultiDataSet` object has been created, we can use it in other packages that have been designed to perform integration analyses. The vast majority of existing R/Bioconductor packages that have been developed for this purpose assumes that the different tables contain the same individuals (i.e. complete cases). This is the case of `omicade4` method. Here, we illustrate how to easily obtain complete cases having a `MultiDataSet` object as input.

### 3.3.1 Interacting with `omicade4`

`omicade4` provides functions for multiple co-inertia analysis as well as for data visualization that will help the interpretation when integrating several omic datasets.

`omicade4` can be loaded by executing:

```
library(omicade4)
```

The function `mcia` was designed to perform multiple co-inertia analysis. It requires a list having the three omic datasets as each component. Herein, we start by illustrating how to perform this analysis in the case of using original `ExpressionSet` and `MethylatioSet` objects. That is, in the case of not using a `MultiDataSet`. By doing that we will illustrate how having a proper way of encapsulating multi-omic data may help downstream analyses.

The function `assayDataElement` can be used to retrieve the matrix having gene expression or methylation levels from an `ExpressionSet` or `MethylationSet`, respectively. Therefore, the required list to be passed through `mcia` function can be obtained by executing:

```
dat <- list(
  assayDataElement(gSet1, "exprs"),
  assayDataElement(gSet2, "exprs"),
  assayDataElement(mSet, "meth")
)
```

Then, this list is used to perform multiple coinertia analysis

```
fit <- mcia(dat)
```

```
## Error in mcia(dat): Nonequal number of individual across data.frames
```

However, an error message is obtained. The error is caused because the matrices have a different number of samples (columns). That is, there are samples not present in all datasets. This could be overcome by writing some code to get tables having the same samples in each matrix. This can be easily addressed by using a `MultiDataSet` object as input because the function `commonSamples` performs the selection of complete cases.

The following code creates a new `MultiDataSet` with common samples

```
md.r <- commonSamples(md)
```

We can verify that the three datasets are having the same samples by executing:

```
lapply(as.list(md.r), dim)
```

```
## $`expression+gSet1`
## [1] 300  12
## 
## $`expression+gSet2`
## [1] 300  12
## 
## $methylation
## [1] 300  12
```

The object `md.r` can be passed through `mcia` as a list to perform multiple co-inertia analysis.

```
fit <- mcia(as.list(md.r))
```

`fit` contains the output of `mcia` function. For more information about `mcia` and the content of `fit` type `?omicade4::mcia` in the R session.

To make this process easier to the user, `MultiDataSet` includes a wrapper to apply `mcia` to a `MultiDataSet` object:

```
fit <- w_mcia(md)
```

```
fit
```

```
## $call
## omicade4::mcia(df.list = as.list(object))
## 
## $mcoa
## Multiple Co-inertia Analysis
## list of class mcoa
## 
## $pseudoeig: 12 pseudo eigen values
## 0.6485 0.4588 0.3645 0.3096 0.2575 ...
## 
## $call: mcoa2(X = ktcoa, scannf = FALSE, nf = cia.nf)
## 
## $nf: 2 axis saved
## 
##    data.frame nrow ncol content                                 
## 1  $SynVar    12   2    synthetic scores                        
## 2  $axis      900  2    co-inertia axis                         
## 3  $Tli       36   2    co-inertia coordinates                  
## 4  $Tl1       36   2    co-inertia normed scores                
## 5  $Tax       12   2    inertia axes onto co-inertia axis       
## 6  $Tco       900  2    columns onto synthetic scores           
## 7  $TL        36   2    factors for Tli Tl1                     
## 8  $TC        900  2    factors for Tco                         
## 9  $T4        12   2    factors for Tax                         
## 10 $lambda    3    2    eigen values (separate analysis)        
## 11 $cov2      3    2    pseudo eigen values (synthetic analysis)
## other elements: Tlw Tcw blo Tc1 RV 
## 
## $coa
## $coa$`expression+gSet1`
## Duality diagramm
## class: nsc dudi
## $call: FUN(df = X[[i]], scannf = FALSE, nf = ..2)
## 
## $nf: 2 axis-components saved
## $rank: 11
## eigen values: 0.0001935 0.0001143 7.572e-05 6.979e-05 5.893e-05 ...
##   vector length mode    content       
## 1 $cw    12     numeric column weights
## 2 $lw    300    numeric row weights   
## 3 $eig   11     numeric eigen values  
## 
##   data.frame nrow ncol content             
## 1 $tab       300  12   modified array      
## 2 $li        300  2    row coordinates     
## 3 $l1        300  2    row normed scores   
## 4 $co        12   2    column coordinates  
## 5 $c1        12   2    column normed scores
## other elements: N 
## 
## $coa$`expression+gSet2`
## Duality diagramm
## class: nsc dudi
## $call: FUN(df = X[[i]], scannf = FALSE, nf = ..2)
## 
## $nf: 2 axis-components saved
## $rank: 11
## eigen values: 0.0002146 0.0001212 9.3e-05 7.467e-05 6.397e-05 ...
##   vector length mode    content       
## 1 $cw    12     numeric column weights
## 2 $lw    300    numeric row weights   
## 3 $eig   11     numeric eigen values  
## 
##   data.frame nrow ncol content             
## 1 $tab       300  12   modified array      
## 2 $li        300  2    row coordinates     
## 3 $l1        300  2    row normed scores   
## 4 $co        12   2    column coordinates  
## 5 $c1        12   2    column normed scores
## other elements: N 
## 
## $coa$methylation
## Duality diagramm
## class: nsc dudi
## $call: FUN(df = X[[i]], scannf = FALSE, nf = ..2)
## 
## $nf: 2 axis-components saved
## $rank: 11
## eigen values: 0.005874 0.005329 0.004701 0.003055 0.002467 ...
##   vector length mode    content       
## 1 $cw    12     numeric column weights
## 2 $lw    300    numeric row weights   
## 3 $eig   11     numeric eigen values  
## 
##   data.frame nrow ncol content             
## 1 $tab       300  12   modified array      
## 2 $li        300  2    row coordinates     
## 3 $l1        300  2    row normed scores   
## 4 $co        12   2    column coordinates  
## 5 $c1        12   2    column normed scores
## other elements: N 
## 
## 
## attr(,"class")
## [1] "mcia"
```

### 3.3.2 Interacting with `iClusterPlus`

`iClusterPlus` was developed for integrative clustering analysis of multi-type genomic data. Hence, `MultiDataSet` is the perfect candidate to be established as the default type of input type.

To start the analysis `iClusterPlus` needs to be loaded:

```
library(iClusterPlus)
```

The typical call to `iClusterPlus`, using our three datasets, should be:

```
fit <- iClusterPlus(
    dt1 = assayDataElement(gSet1, "exprs"),
    dt2 = assayDataElement(gSet2, "exprs"),
    dt3 = assayDataElement(mSet, "meth"),
    type = c("gaussian", "gaussian", "gaussian"),
    lambda = c(0.04,0.61,0.90),
    K = 5,
    maxiter = 10
)
```

We refer the reader to the help of `iClusterPlus` and to the manual entitled *“iClusterPlus: integrative clustering of multiple genomic data sets”* for more information about the arguments `lambda`, `K` and `maxiter`. This document will only shortly cover `dt*` and `type` arguments.

In this case, the package can deal with tables having a different number of samples. To make the call to `iClusterPlus` function easier for the user, `MultiDataSet` includes a wrapper to apply `iClusterPlus` to a `MultiDataSet` object. This method automatically creates the argument `type` required by `iClusterPlus`. This argument is created by taking into account the type of datasets included in `MultiDataSet` object. In particular:

- The type assigned to `ExpressionSet` is *gaussian*
- The type assigned to `MethylationSet` is *gaussian*
- The type assigned to `SnpSet` is *multinomial*

The other arguments accepted by `iClusterPlus` can be given to the function through the wrapper:

```
fit <- w_iclusterplus(
  md,
  lambda = c(0.04,0.61,0.90),
  K = 5,
  maxiter = 10
)
```

The obtained result is homologous to the previous version of `iClusterPlus` call. Both of them can be plotted as suggested in `iClusterPlus`’s vignette.

```
library(lattice) # Required for iClusterPlus's plotHeatmap
library(gplots) # Required to generate the colour-schemes

col.scheme <- alist()
col.scheme[[1]] <- bluered(256) # colours for gSet1
col.scheme[[2]] <- bluered(256) # colours for gSet2
col.scheme[[3]] <- greenred(256) # colours for mSet

plotHeatmap(fit=fit,datasets=lapply(as.list(commonSamples(md)), t),
  type=c("gaussian","gaussian","gaussian"), col.scheme = col.scheme,
  row.order=c(T,T,T),sparse=c(T,F,T),cap=c(F,F,F)
)
```

In this case, the first two rectangles correspond to the two `ExpressionSet`s while the last rectangle to the `MethylationSet`.

# 4 Summary and Conclusion

This document aimed to illustrate how `MultiDataSet` can be used jointly with third party R/Bioconductor packages implementing methods for data analysis and integration. The first part of the document describes how to create `MultiDataSet` objects and how the functions `add_genexp` and `add_methy` are used to add gene expression and methylation data. The second part deals with the use of `MultiDataSet` objects and the use of `mcia` function of `omicade4` and `iClusterPlus`.

The current version of `MultiDataSet` supports two downstream analyses: *iCluster* (by Shen 2009) implemented in `iClusterPlus` R package and *multiple co-inertia analysis* (by Bady 2004) implemented in `omicade4`. The appendix of the document includes the code we wrote for developing the wrappers for `mcia` and `iClusterPlus` (also included in `MultiDataSet` R package). As can be seen, the code takes profit of `MultiDataSet` features and it is very easy to understand. We are working on developing more wrappers for integrative and data analysis that will be incorporated in future versions of `MultiDataSet`.

# 5 Appendix: Wrappers

## 5.1 Wrapper for `omicade4`

The following is the definition of the wrapper include in `MultiDataSet` for `omicade4`’s function `mcia`. It follows the steps explained in the main document.

```
setMethod(
    f = "w_mcia",
    signature = "MultiDataSet",
    definition = function(object, ...) {
        # Obtain the common samples between datasets
        object <- commonSamples(object)
        
        # Call mcia with the generated arguments and the user's arguments
        omicade4::mcia(as_list(object), ...)
    }
)
```

## 5.2 Wrapper for `iClusterPlus`

The following is the code written to create the wrapper for `iClusterPlus`, that allows using a `MultiDataSet` object as input. The method has three parts:

1. It used the function `commonSamples` to reduce the amount of samples included in each dataset to the common samples along the datasets. This is a way to speed-up `iClusterPlus`. The argument `commonSamples`, by default set to `TRUE`, allows changing this feature.
2. It creates the argument `type` requested by `iClusterPlus` by using the name of each dataset. Here we consider that *gene expression* as gaussian, *methylation* as gaussian and *snps* as multinomial.
3. Apply `iCluserPlus` by accepting other argument saved into `...`

```
setMethod(
    f = "w_iclusterplus",
    signature = "MultiDataSet",
    definition = function(object, commonSamples=TRUE, ...) {
        if(length(object) > 4) {
            stop("'iClusterPlus' only allows four datasets.")
        }
        
        # Obtain the common samples between datasets
        if(commonSamples) {
            object <- commonSamples(object)
        }
        
        # Put the names iClusterPlus requires on the datasets
        datasets <- lapply(as.list(object), t)
        names(datasets) <- paste("dt", 1:length(datasets), sep="")
        
        # Generate the "type" argument for iClusterPlus
        datasets[["type"]] <- sapply(names(object), function(nm) {
            ifelse(startsWith(nm, "expression"), "gaussian",
                   ifelse(startsWith(nm, "methylation"), "gaussian", "multinomial"))
        })
        names(datasets[["type"]]) <- paste("dt", 1:(length(datasets) -1), sep="")
        
        # Call iClusterPlus with the generated arguments and the user's arguments
        do.call("iClusterPlus", c(datasets, list(...)))
    }
)
```
