# Supplementary figures and images for: MultiDataSet: an R package for encapsulating multiple data sets with application to omic data integration
Source: BMC Bioinformatics. 2017 Jan 17;18:36. doi: 10.1186/s12859-016-1455-1 (PMC5240259; doi:10.1186/s12859-016-1455-1)

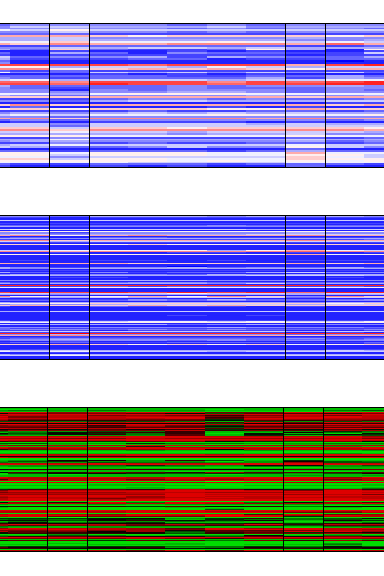

Supplement: Additional file 1: — “Using MultiDataSet with third party R packages”. This file illustrates how to perform an integration analysis using multivariate co-inertia analysis (omicade4) and clustering of multiples tables (iClusterPlus). (ZIP 38 kb) [file 12859_2016_1455_MOESM1_ESM.zip › sm1_MultiDataSet_UseCase_3party_files/figure-html/iclusterplus_show-1.png]
