# Supplementary material for: MultiDataSet: an R package for encapsulating multiple data sets with application to omic data integration
Source: BMC Bioinformatics. 2017 Jan 17;18:36. doi: 10.1186/s12859-016-1455-1 (PMC5240259; doi:10.1186/s12859-016-1455-1)
Supplement: Additional file 2: — “Integration of methylation and expression using MultiDataSet”. This file is a tutorial of how to develop a function to implement a new method using MultiDataSet capabilities. (HTML 78 kb) [file 12859_2016_1455_MOESM2_ESM.html]

Integration of methylation and expression using MultiDataSet


# Integration of methylation and expression using MultiDataSet

#### *Carles Hernandez-Ferrer, Carlos Ruiz-Arenas, Alba Beltran-Gomila, Juan R. González*

#### *2016-10-04*

#### Package version: MultiDataSet 1.1.6

# Contents

- 1 Introduction
- 2 Objective
- 3 Implementation
  - 3.1 Function definition
  - 3.2 Data checking
  - 3.3 Preparation of data
  - 3.4 Implementation of the algorithm
  - 3.5 Formatting the results
  - 3.6 Final result
- 4 Application
- References

# 1 Introduction

Projects in biomedicine are generating information about multiple types of omics data. Most of these datasets are publicly available through on-line data backs like GEO (*Gene Expression Omnibus*), TCGA (*The Cancer Genome Atlas*) or EGA (*European Genome-phenome Archive*) among others. Standard association analyses between a given phenotype and a single omic dataset (aka. SNPs, gene expression, methylations,…) are insufficient to address most of the scientific questions that rely on knowing the mechanisms involved in complex diseases. Methods to properly integrate multi-data sets have to be used to analyze such amount of information.

One of the computational challenges when dealing with multi-omic data is to define an easy way to compact multiple pieces of information. This methodology must allow retrieving data for common-samples, subsetting individuals having a specific characteristic or selecting features from a given genomic region or a selected gene. `MultiDataSet` is conceived as a container and manager of multiple omic datasets while allowing performing common object operations such as accession to elements or subsetting among others. The vignette of `MultiDataSet` describes how to manipulate a MultiDataSet object as well as how to perform other basic operations.

In this document, we show how to implement a new integration function using MultiDataSet. The process is exemplified with a function that integrates methylation and expression, which can be found in MEAL package. We propose a general scheme that could be used for any integration function.

# 2 Objective

The objective of this document is to illustrate how to create an omic data integration function taking profit of `MultiDataSet` features. This tutorial is meant for software developers who have created a statistical method to analyze multiple omic datasets and want to implement it using `MultiDataSet` objects as input.

# 3 Implementation

We will exemplify the implementation of an integration algorithm with a function that integrates methylation and expression. This example function can be found in MEAL package (Ruiz, Hernandez-Ferrer, and González 2016) and it is an adaptation of an existing method described in a previous paper (Steenaard et al. 2015). Briefly, the method consists on pairing CpGs to nearby expression probes and to measure the correlation between CpG and expression probes in each pair.

A typical integration function requires 4 parts:

1. Check the parameters
2. Create the appropriate data structures
3. Run the integration algorithm
4. Give a suitable format to the output

The following sub-sections contain the code and an explanation of how to write the 4 parts of an integration function. The complete definition of the function can be found at the end of this section.

## 3.1 Function definition

Before starting with the body of our function, we need to set the parameters. Our function will be called `correlationMethExprs` and will have 11 arguments:

```
correlationMethExprs <- function(multiset, 
                                 meth_set_name = NULL, exprs_set_name = NULL,
                                 vars_meth = NULL, vars_exprs = NULL, 
                                 vars_meth_types = rep(NA, length(vars_meth)), 
                                 vars_exprs_types = rep(NA, length(vars_exprs)),
                                 sel_cpgs, flank = 250000, num_cores = 1, verbose = TRUE){
```

`multiset` is the `MultiDataSet` containing the data. `meth_set_name` and `exprs_set_name` are the names of the methylation and expression datasets in `multiset` object. The arguments `vars_meth` and `vars_exprs` denotes the variables used to adjust linear models between gene expression and methylation (e.g. sex, age, …). The arguments `vars_meth_types` and `vars_exprs_types` encodes the type of variables we are adjusting for. The argument `sel_cpgs` is used to select those CpGs we are interested in analyzing. The argument `flank` indicates the minimum distance to pair a CpG to an expression probe. Finally, `num_cores` will be used to allow parallelizing the algorithm and `verbose` is a flag to decide whether display messages during the executing. In the following subsections, these arguments will be further discussed and explained.

## 3.2 Data checking

When users work with a package they could easily pass unsuitable objects to the function (e.g. a `data.frame` with misspelled column names). If our function does not detect these mistakes, their inner processes may throw an error which will be very difficult to understand by the users. Consequently, they will not be able to fix it and run the function properly. A good practice to avoid these problems is to introduce data checks to the input object to ensure that the function could run properly. If there are problems, it returns an error with the mistake and, sometimes, proposes ways to solve it.

When creating integration functions with `MultiDataSet`, data checking is performed at two levels. On one hand, functions that add data to `MultiDataSet` check that the introduced datasets fulfill some requirements. In our example, we will use the functions of `MultiDataSet` package to add methylation and expression that already includes checks for the incoming datasets. For more information on how to create a function to add new classes to a `MultiDataSet` take a look at “Adding a new type of data to MultiDataSet objects” (Supplementary Material 3).

On the other hand, integration functions must do other checks to the incoming `MultiDataSet`. In our function, we will include three checks. The first is just to check that our object is a `MultiDataSet`. Second, we will check that `meth_set_name` and `exprs_set_name` are characters or NULL. These parameters are useful to choose the datasets used in the analysis when we have several methylation or expression sets. If these parameters have the right format, we will check that our `MultiDataSet` contains datasets with these names and that these datasets have a `GenomicRanges`. Finally, we will check that the parameter `flank` is a numeric that contains only one positive number.

```
# [Check 1] Is multiset a MultiDataSet object?
if (!is(multiset, "MultiDataSet")){
    stop("multiset must be a MultiDataSet")
}

# [Check 2A] Are meth_set_name and exprs_set_name characters?
if (!(is.character(meth_set_name) | is.null(meth_set_name))){
    stop("meth_set_name must be a character.")
} 
if (!(is.character(exprs_set_name) | is.null(exprs_set_name))){
    stop("exprs_set_name must be a character.")
} 
    
# Add the dataset type to the names
meth_set_name <- paste(c("methylation", meth_set_name), collapse = "+")
exprs_set_name <- paste(c("expression", exprs_set_name), collapse = "+")


# [Check 2B] Has multiset the right sets?
if (!all(c(meth_set_name, exprs_set_name) %in% names(multiset))){
    stop("multiset must contain meth_set_name and exprs_set_name.")
}

if (!all(c(meth_set_name, exprs_set_name) %in% rowRangesElements(multiset))){
    stop("multiset must contain meth_set_name and exprs_set_name.")
}

# [Check 3] Is flank numeric, positive and has only one number?
if (!is(flank, "numeric") || length(flank) > 1 || flank < 0){
    stop("flank must be a positive integer")
}
```

It is important to notice the lines where we add the dataset type to the set names. When users add a set to a `MultiDataSet`, the name of the incoming set includes its data type. Consequently, all methylation objects start by “methylation” and all expression sets by “expression”. This procedure reduces the checks in the integration function. Using the name of the `MultiDataSet` datasets, we can deduce their original class. Then, we can be sure that these datasets already have some specific features that we do not need to check again.

## 3.3 Preparation of data

`MultiDataSet` is useful for managing omic datasets but the implementation of the algorithm requires using simpler data structures. For example, imagine that we want to run a simpler linear regression. R has implemented in its base package the function `lm`, which requires `data.frame`s or `list`s. Therefore, the first step would be transforming the data of `MultiDataSet` to a data structure compatible with the functions that we will call. In this subsection, we will exemplify how to perform this process. We will show how to obtain the classes required by the functions that perform the algorithm: `ExpressionSet`, `MethylationSet`, and `GenomicRanges`.

Our algorithm requires that all the datasets have the same samples. Consequently, the first step is to filter the datasets to contain only those samples present in both datasets. This step can be easily done using the method `commonSamples`:

```
# Select only our datasets
multiset <- multiset[ , c(meth_set_name, exprs_set_name)]

## Select common samples
multiset <- commonSamples(multiset)
```

We can select specific datasets of `MultiDataSet` using `[` and pass their names to the second argument. This code returns a `MultiDataSet` with the specified datasets. The method `commonSamples` returns a `MultiDataSet` where all datasets have the same samples in the same order. Given that `multiset` can have other datasets, it is advisable to previously reduce it to only the datasets that we will use. Otherwise, we might lose samples that are not present in other datasets.

Next, we will get the original `MethylationSet` and `ExpressionSet` from `multiset`. The original classes will help us to perform easily perform other operations that we will do later. The `[[` operator retrieves the original datasets from a `MultiDataSet`:

```
# Obtain the original objects
mset <- multiset[[meth_set_name]]
eset <- multiset[[exprs_set_name]]
```

In this kind of analysis, it is very common to use only the CpGs that in a previous analysis had been found differentially methylated. We have included the parameter `sel_cpgs` to select CpGs:

```
if (!missing(sel_cpgs)){
    if (!is.character(sel_cpgs) | length(sel_cpgs) == 0){
        stop("sel_cpgs must be a character vector with the name of the CpGs to be used.")
    } else{
        mset <- mset[sel_cpgs, ]
    }
}
```

This parameter is not mandatory, so the first line checks if it is present. The second line ensures that the parameter contains the names of CpGs in a character vector.

Finally, we get the `GenomicRanges` of the methylation and expression sets, with `rowRanges` accessors:

```
# Get GRanges
rangesMeth <- rowRanges(multiset)[[meth_set_name]]
rangesMeth <- rangesMeth[featureNames(mset)]
rangesExprs <- rowRanges(multiset)[[exprs_set_name]]
```

## 3.4 Implementation of the algorithm

The integration algorithm that we will use consists of three steps:

- Pair CpGs to close expression probes.
- Remove co-variables effect (this step is optional).
- Compute the association between expression and methylation.

To pair the CpGs and the expression probes, we will use the following method. For each CpG, we will define a region of interest using the position of the CpG plus-minus the parameter `flank` (`flank` units are bases: 250Kb by default). Then, we will pair this CpG to all the expression probes whose start and end coordinates are inside the region, using the function `findOverlaps` of `GenomicRanges` package. If there are no CpG-expression probes pairs, an empty data.frame is returned and a warning is thrown:

```
start(rangesMeth) <- start(rangesMeth) - flank
end(rangesMeth) <- end(rangesMeth) + flank
pairs <- GenomicRanges::findOverlaps(rangesExprs, rangesMeth,  type = "within")
pairs <- data.frame(cpg = rownames(mset)[S4Vectors::subjectHits(pairs)], 
                    exprs = rownames(eset)[S4Vectors::queryHits(pairs)], 
                    stringsAsFactors = FALSE)

# If there are no pairs, do not continue
if (nrow(pairs) == 0){
    warning("There are no expression probes in the range of the cpgs. An empty data.frame will be returned.")
    return(data.frame(cpg = character(0), exprs = character(0), Beta = integer(0), 
                      se = integer(0), P.Value = integer(0), adj.P.val = integer(0)))
}
```

Now, we will introduce the possibility of removing the effect of co-variates. We will perform a linear regression of our data against the co-variables and we will compute the residuals. Then, these residuals will be used to compute the association. We have defined the function `setResidues` to do this process taking profit of `eSet` derived objects. Although its definition can be found in the complete integration function, an exhaustive explanation will not be provided.

Four additional parameters of the integration function are needed here. `vars_meth` and `vars_meth_type` contain the name and the type of the methylation co-variables. `vars_exprs` and `vars_exprs_types` are the equivalent for expression:

```
# Computing residuals
methres <- setResidues(mset, vars_names = vars_meth, vars_types = vars_meth_types)
exprsres <- setResidues(eset, vars_names = vars_exprs, vars_types = vars_exprs_types)
```

The last step is to compute the association between each CpG-expression probe pair using a linear regression, where expression will be the outcome. We have implemented this step using the `lm` function and `mclapply` of parallel package. This design allows the user to parallelize this step:

```
## We define the function to be used in the mclapply
residualsCorr <- function (methy_res, exprs_res){
    fit <- lm(exprs_res ~ methy_res)
    return(c(summary(fit)$coef[2, 1], summary(fit)$coef[2,2], summary(fit)$coef[2,4]))
}

# use mclapply to speed up computation
regvals <- mclapply(1:nrow(pairs), 
                    function(x) residualsCorr(methres[pairs[x, 1], ], exprsres[pairs[x, 2], ]), 
                    mc.cores = num_cores)
```

## 3.5 Formatting the results

The very last step is to generate an output suitable for users. In our case, the results will be returned in a `data.frame` containing the names of the CpGs and expression probes and the estimates of the linear regression:

```
res <- data.frame(pairs, t(data.frame(regvals)))
colnames(res) <- c("cpg", "exprs", "Beta", "se", "P.Value")
res$adj.P.Val <- p.adjust(res$P.Value, "BH")
res <- res[order(res$adj.P.Val), ]
rownames(res) <- NULL
res
```

## 3.6 Final result

This integration function was previously implemented in MEAL package. The next chunk contains the whole function as it can be found in the package. It should be noticed that there are lines that have not been previously commented, such as code to display messages during the execution:

```
correlationMethExprs <- function(multiset, 
                                 meth_set_name = NULL, exprs_set_name = NULL,
                                 vars_meth = NULL, vars_exprs = NULL, 
                                 vars_meth_types = rep(NA, length(vars_meth)), 
                                 vars_exprs_types = rep(NA, length(vars_exprs)),
                                 sel_cpgs, flank = 250000, num_cores = 1, verbose = TRUE){
    
    ######################################################################################
    ## Data Checking
    # Check object is a MultiDataSet
    if (!is(multiset, "MultiDataSet")){
        stop("multiset must be a MultiDataSet")
    }
    
    # Check meth_set_name and exprs_set_name are characters
    if (!(is.character(meth_set_name) | is.null(meth_set_name))){
        stop("meth_set_name must be a character.")
    } 
    if (!(is.character(exprs_set_name) | is.null(exprs_set_name))){
        stop("exprs_set_name must be a character.")
    } 
            
    # Add the dataset type to the names
    meth_set_name <- paste(c("methylation", meth_set_name), collapse = "+")
    exprs_set_name <- paste(c("expression", exprs_set_name), collapse = "+")
    
    
    # Check our object has the right sets
    if (!all(c(meth_set_name, exprs_set_name) %in% names(multiset))){
        stop("multiset must contain meth_set_name and exprs_set_name.")
    }
    
    if (!all(c(meth_set_name, exprs_set_name) %in% rowRangesElements(multiset))){
        stop("multiset must contain meth_set_name and exprs_set_name.")
    }
    
    # Check that flank is numeric, positive and is only one number
    if (!is(flank, "numeric") || length(flank) > 1 || flank < 0){
        stop("flank must be a positive integer")
    }
    #####################################################################################
    
    #####################################################################################
    ## Preparation of data
    #############################
    ## Preparation of data 1
    # Select only our sets
    multiset <- multiset[, c(meth_set_name, exprs_set_name)]
    
    ## Select common samples
    multiset <- commonSamples(multiset)
    #############################
    ## Preparation of data 2

    mset <- multiset[[meth_set_name]]
    eset <- multiset[[exprs_set_name]]
    
    #############################
    ## Preparation of data 3
    if (!missing(sel_cpgs)){
        if (!is.character(sel_cpgs) | length(sel_cpgs) == 0){
            stop("sel_cpgs must be a character vector with the name of the CpGs to be used.")
        } else{
            mset <- mset[sel_cpgs, ]
        }
    }

    #############################
    ## Preparation of data 4
    # Compute Methylation-Expression pairs
    rangesMeth <- rowRanges(multiset)[[meth_set_name]]
    rangesMeth <- rangesMeth[featureNames(mset)]
    rangesExprs <- rowRanges(multiset)[[exprs_set_name]]
    #####################################################################################
    
    #####################################################################################
    ## Implementation of the algorithm
    #############################
    ## Implementation of the algorithm 1
    start(rangesMeth) <- start(rangesMeth) - flank
    end(rangesMeth) <- end(rangesMeth) + flank
    pairs <- GenomicRanges::findOverlaps(rangesExprs, rangesMeth,  type = "within")
    pairs <- data.frame(cpg = rownames(mset)[S4Vectors::subjectHits(pairs)], 
                        exprs = rownames(eset)[S4Vectors::queryHits(pairs)], 
                        stringsAsFactors = FALSE)
    if (nrow(pairs) == 0){
        warning("There are no expression probes in the range of the cpgs. An empty data.frame will be returned.")
        return(data.frame(cpg = character(0), exprs = character(0), Beta = integer(0), 
                          se = integer(0), P.Value = integer(0), adj.P.val = integer(0)))
    }
    #############################
    ## Filter sets to only features in the pairs
    mset <- mset[unique(pairs[ , 1]), ]
    eset <- eset[unique(pairs[ , 2]), ]
    
    
    if (verbose){
        message("Computing residuals")
    }
    
    #############################
    ## Implementation of the algorithm 2
    # Computing residuals
    methres <- setResidues(mset, vars_names = vars_meth, vars_types = vars_meth_types)
    exprsres <- setResidues(eset, vars_names = vars_exprs, vars_types = vars_exprs_types)
    #############################
    if (verbose){
        message("Computing correlation Methylation-Expression")
    }
    
    #############################
    ## Implementation of the algorithm 3
    residualsCorr <- function (methy_res, exprs_res){
        fit <- lm(exprs_res ~ methy_res)
        return(c(summary(fit)$coef[2, 1], summary(fit)$coef[2,2], summary(fit)$coef[2,4]))
    }
    regvals <- mclapply(1:nrow(pairs), 
                    function(x) residualsCorr(methres[pairs[x, 1], ], exprsres[pairs[x, 2], ]), 
                    mc.cores = num_cores)
    #####################################################################################

    #####################################################################################
    ## Foprmatting the results
    res <- data.frame(pairs, t(data.frame(regvals)))
    colnames(res) <- c("cpg", "exprs", "Beta", "se", "P.Value")
    res$adj.P.Val <- p.adjust(res$P.Value, "BH")
    res <- res[order(res$adj.P.Val), ]
    rownames(res) <- NULL
    res
    #####################################################################################
}

## Function to remove covariables effect of methylation and expression data
setResidues <- function(set, vars_names, vars_types){
    if (length(vars_names) != 0){
        if (ncol(pData(set)) == 0 | nrow(pData(set)) == 0){
            warning("set has no phenotypes. No residues will be computed")
        } else if (!sum(vars_names %in% colnames(pData(set)))){
            if (is(set, "ExpressionSet")){
                warning("vars_exprs is/are not a valid column of the eset phenoData. No residues will be computed")
            } else{
                warning("vars_meth is/are not a valid column of the mset phenoData. No residues will be computed")
            }
        }else{
            pData(set) <- preparePhenotype(phenotypes = pData(set), 
                                           variable_names = vars_names,
                                           variable_types = vars_types)
            model <- createModel(data = pData(set))
            if (is(set, "ExpressionSet")){
                vals <- exprs(set)
            } else if (is(set, "RatioSet")){
                vals <- minfi::getBeta(set)
            } else{
                vals <- betas(set)
                ## Convert methylation to M values prior fitting the linear model
                vals <- minfi::logit2(vals)
            }
            res <- residuals(limma::lmFit(vals, model), vals)
            if (is(set, "MethylationSet")){
                res <- minfi::ilogit2(res)
            }
            return(res)
        }
    }
    if (is(set, "ExpressionSet")){
        return(exprs(set))
    } else if (is(set, "RatioSet")){
        return(minfi::getBeta(set))
    } else{
        return(betas(set))
    }
}
```

# 4 Application

Now, it is time to check that the function is working. To this end, we will use MEALData package that contains methylation and expression data from the same set of samples. The following lines load the libraries used in our function:

```
library(MultiDataSet)
library(GenomicRanges)
library(MEALData)
library(limma)
library(parallel)
library(minfi)
```

MEALData contains the expression data in an `ExpressionSet`. However, the methylation data has the beta values in a matrix and the phenotypic data in a data.frame. Therefore, we will create a `MethylationSet` and then we will add the `ExpressionSet` and the `MethylationSet` to a `MultiDataSet`:

```
meth <- prepareMethylationSet(betavals, pheno)
multi <- createMultiDataSet()
multi <- add_methy(multi, meth)
fvarLabels(eset)[1] <- "chromosome" 
multi <- add_genexp(multi, eset)
```

We are ready to use our function. To speed up the computation, we will select three CpGs to do the pairing:

```
res <- correlationMethExprs(multi, sel_cpgs =  c("cg17504453", "cg25946965", "cg07938442"))
res
```

```
##           cpg        exprs        Beta        se     P.Value adj.P.Val
## 1  cg07938442 ILMN_1669714  0.76128677 0.2794841 0.008630436 0.1985000
## 2  cg07938442 ILMN_1702105 -1.82958179 1.1971030 0.132159072 0.6079317
## 3  cg07938442 ILMN_1748738 -0.53573965 0.3386233 0.119358329 0.6079317
## 4  cg17504453 ILMN_2072178  5.57719204 3.2693201 0.093665133 0.6079317
## 5  cg17504453 ILMN_2128639  2.84632500 1.4966575 0.062441577 0.6079317
## 6  cg07938442 ILMN_1737168  0.58850826 0.4907462 0.235586957 0.6888160
## 7  cg07938442 ILMN_1784515  0.32768625 0.2723331 0.234031285 0.6888160
## 8  cg07938442 ILMN_2401884 -0.34216922 0.2878040 0.239588168 0.6888160
## 9  cg07938442 ILMN_1775373 -0.22365734 0.2167502 0.306650716 0.7067769
## 10 cg17504453 ILMN_2383693  0.80835009 0.7844390 0.307294317 0.7067769
## 11 cg07938442 ILMN_2276598 -0.24453166 0.2806600 0.387392017 0.8100015
## 12 cg17504453 ILMN_1679417 -0.19293467 0.4756460 0.686590693 0.8525235
## 13 cg07938442 ILMN_1720243 -0.08213272 0.2904258 0.778391005 0.8525235
## 14 cg07938442 ILMN_1725726  0.21801928 0.3828479 0.571357755 0.8525235
## 15 cg07938442 ILMN_1746561 -0.66999817 1.1575229 0.565073206 0.8525235
## 16 cg07938442 ILMN_1754179 -0.49924395 0.9492902 0.601062944 0.8525235
## 17 cg07938442 ILMN_1758829  0.09016861 0.2596968 0.729760728 0.8525235
## 18 cg07938442 ILMN_1759154 -0.72586539 1.5029781 0.631047126 0.8525235
## 19 cg07938442 ILMN_1765248 -0.14994781 0.2627212 0.570494635 0.8525235
## 20 cg07938442 ILMN_2384857  0.15218333 0.3488957 0.664409384 0.8525235
## 21 cg07938442 ILMN_2401883 -0.07131496 0.2280413 0.755669892 0.8525235
## 22 cg17504453 ILMN_1739283 -0.22362565 2.0792906 0.914744815 0.9563241
## 23 cg07938442 ILMN_1653200  0.02834706 1.3791744 0.983676103 0.9836761
```

`res` is a `data.frame` with the results of the algorithm.

# References

Ruiz, C, C Hernandez-Ferrer, and J González. 2016. “MEAL: Perform methylation analysis. R package version 1.2.3.”

Steenaard, Rebecca V, Symen Ligthart, Lisette Stolk, Marjolein J Peters, Joyce B van Meurs, Andre G Uitterlinden, Albert Hofman, Oscar H Franco, and Abbas Dehghan. 2015. “Tobacco smoking is associated with methylation of genes related to coronary artery disease.” *Clinical Epigenetics* 7 (1): 54. doi:10.1186/s13148-015-0088-y.
