# Supplementary material for: MultiDataSet: an R package for encapsulating multiple data sets with application to omic data integration
Source: BMC Bioinformatics. 2017 Jan 17;18:36. doi: 10.1186/s12859-016-1455-1 (PMC5240259; doi:10.1186/s12859-016-1455-1)
Supplement: Additional file 3: — “Adding a new type of data to MultiDataSet objects”. This file exemplifies how to create a method to incorporate data from S4 classes to a MultiDataSet object. (HTML 66 kb) [file 12859_2016_1455_MOESM3_ESM.html]

Adding a new type of data to MultiDataSet objects


# Adding a new type of data to MultiDataSet objects

#### *Carles Hernandez-Ferrer, Carlos Ruiz-Arenas, Alba Beltran-Gomila, Juan R. González*

#### *2016-10-04*

#### Package version: MultiDataSet 1.1.6

# Contents

- 1 Introduction
- 2 Objective
- 3 Implementation
  - 3.1 Defining ProteomeSet
  - 3.2 Loading Proteome Data
  - 3.3 Extending MultiDataSet
  - 3.4 Data example: Adding Proteome data to MultiDataSet objects

# 1 Introduction

Recent advances in biotechnology are introducing new sources of biological information. As a result, developers need to create classes to properly storage and manage these new kinds of data.

`MultiDataSet` has methods to deal with three common datasets: gene expression, SNPs data, and DNA methylation. Gene expression from an `ExpressionSet` can be added to a `MultiDataSet` using `add_genexp` (for microarrays) or `add_rnaseq` (for RNAseq) functions. SNP data can be incorporated into a `MultiDataSet` by using the function `add_snp`. DNA methylation encapsulated in a `MethylationSet` object can be added into a `MultiDataSet` using `add_methy`. In addition, `MultiDataSet` is also able to work with any other class of objects based on `eSet` or `SummarizedExperiment`, two general classes of Bioconductor framework. Consequently, developers can implement methods to expand `MultiDataSet` to work with their own classes.

In this document, we show how to create a new method to add a new class of objects into `MultiDataSet`. This process is exemplified by creating a new class to store proteomic data. To this end, we will extend the `eSet` class. It should be noticed that the process would be very similar if the class was based on `SummarizedExperiment`.

# 2 Objective

The objective of this document is to illustrate how to create a method to add a new class of object to `MultiDataSet`. This tutorial is meant for software developers who have developed a new class to manage any new omic data or another type of information to be included in `MultiDataSet` objects.

# 3 Implementation

Proteome data is commonly represented as a matrix of protein’s levels. This data has a special characteristic: some of the information cannot be measure due to the limit of detection (LOD) and limit of quantification (LOQ). Having LOD information in the data matrix can be crucial when performing statistical analyses. Considering proteins as the outcome requires using different statistical methods than those used for analyzing, for instance, gene expression data. LOD makes that proteins are left-censored variables making impossible the use of standard methods of analysis such as linear regression or t-test.

One approach that can be adopted to deal with this type of data is to assign LOD/2 to those values that are below LOD. This will allow the user to analyze protein data using standard packages such as `limma`, which uses linear models. However, this approach is biased. Other methods have been developed to properly analyze left-censored variables. Those methods require knowing the LOD of each protein. Therefore, having information about both protein’s levels and LOD is crucial for downstream analyses. Currently, a class that stores protein data with LOD does not exist. To solve this issue, we propose to create `ProteomeSet`, a new class based on `eSet`. Our new class will contain the raw protein levels, information about LOD and protein levels having data below LOD equal to LOD/2.

We will begin by defining the new class: `ProteomeSet`. Second, we will develop a function to load the protein data and the LOD into R and to create our `ProteomeSet`. Third, we will implement a method for `MultiDataSet` to add `ProteomeSet`s. Finally, we will show the application of the code by creating a `MultiDataSet` with protein data.

## 3.1 Defining ProteomeSet

We have chosen to extend `eSet` to implement our `ProteomeSet` because we can take profit of `eSet`s’ methods and structure. Therefore, our `ProteomeSet` will also have the phenotype and feature data as well as methods to retrieve data. Given that `eSet` is defined in Biobase package, we should load it prior to the definition of `ProteomeSet`:

```
library(Biobase)
setClass (
    Class = "ProteomeSet",
    contains = "eSet", 
    prototype = prototype(new("VersionedBiobase",
                              versions = c(classVersion("eSet"), ProteomeSet = "1.0.0")))
)
```

This `ProteomeSet` is defined as another `eSet` object. As previously mentioned, proteome data should contain some specific features. The `setValidity` function defines the requirements that an object must accomplish to be valid. assayData of `ProteomeSet` objects should have two slots to encapsulate both raw and modified data (e.g. values below LOD replaced by LOD/2). These slots will be called `raw` and `prots`, respectively. `ProteomeSet` should also contain information about LOD and LOQ. This data will be obtained from the columns `LoD.T` and `LoD.B` available as featureData. We can introduce these requirements with the following lines of code:

```
setValidity("ProteomeSet", function(object) {
    
    ## Check that object has the slots 'prots' and 'raw' in assayData
    msg <- validMsg(NULL, assayDataValidMembers(assayData(object), c("prots", "raw")))
    
    ## Check that objects has the columns 'LoD.T' and 'LoD.B' in featureData
    msg <- validMsg(msg, ifelse(any(!c("LoD.T", "LoD.B") %in% fvarLabels(object)), "fData must contain columns 'LoD.T' and 'LoD.B'", FALSE))
    if (is.null(msg)){
        TRUE
    } else{
        msg
    }
})
```

In this subsection, we have covered the essentials of extending a class based on `eSet`. Readers interested in more advanced features can find more information about extending R classes and extending eSets.

## 3.2 Loading Proteome Data

Here, we create a function that loads proteome data from a text file, replaces values below LOD and above LOQ and returns a `ProteomeSet` with the available data. Correction of *limit of detection* is commonly defined as follows:

1. If the expression of a protein is below its LOD, it is replaced by LOD/2.
2. If the expression of a protein is above its LOQ, it is replaced by LOQ\*1.5.

The function `read_ldset` will perform this task. It requires four arguments:

- `assayFile`: (character) A path to the proteome’ measurements file.
- `phenoFile`: (character) A path to the samples’ phenotype file.
- `featureFile`: (character) A path to the features’ annotation file.
- `sep`: (character) Indicates the field separator character of the three files above.

The three input files need to be TSV style (TSV: tab-separated file) and must include a header. `assayFile` and `phenoFile` must have a column called `sample` with a unique sample id. `featureFile` must have a column called `feature` with the unique feature id, that must be equal to `assayFile`’s columns names. Moreover, `featureFile` must have two columns called `LoD.B` and `LoD.T`, corresponding to the LOD (*bottom limit of detection*) and the LOQ (*top limit of detection*) of each protein.

`read_lds` checks that the features’ names are the same in assay data and in feature data. It also checks that feature data has the two columns for the *limits of detection*. After performing the two checks, `read_ldset` creates the matrix with the updated level of expression of each protein. Then a `ProteomeSet` is created, containing the raw matrix as `raw` and the updated matrix as `prots`. The phenotypic data and feature’s annotations are also included:

```
read_ldset <- function(assayFile, phenoFile, featureFile, sep="\t") {
    ## Load the threes files that will be used to create the ProteomeSet
    adata <- read.delim(assayFile, sep=sep, header=TRUE, row.names="sample")
    pdata <- read.delim(phenoFile, sep=sep, header=TRUE, row.names="sample")
    fdata <- read.delim(featureFile, sep=sep, header=TRUE, row.names="feature")
    ## /
    
    ## Check that proteins in assay data are the same in feature data
    if(!identical(colnames(adata), rownames(fdata))) {
        stop("Features names in assay data (columns) are not equal to ",
             "features names in feature data (rownames).")
    }
    ##/
    
    ## Check that feature data include columns LoD.B and LoD.T
    if(sum(c("LoD.T", "LoD.B") %in% colnames(fdata)) != 2) {
        stop("Feature data must contain two columns labeled 'LoD.T' (top ",
             "limit of dectection) and 'LoD.B (bottom limit of dectection)")
    }
    ## /
    
    ## Perform the transformation of the protein level of expression
    low <- fdata[colnames(adata), "LoD.B"]
    up <- fdata[colnames(adata), "LoD.T"]
    names(low) <- names(up) <- rownames(fdata)
    faux <- function(x, low, up) {
        x[x < low] <- as.double(low / 2)
        x[x > up] <- as.double(up * 1.5)
        x
    }
    tadata <- mapply(FUN = faux, x = as.list(adata), low = as.list(low), up = as.list(up))
    dimnames(tadata) <- dimnames(adata)
    ## /
    
    ## Create the ExpressionSet with the two matrices
    prot <- new("ProteomeSet",
                assayData = assayDataNew("environment", prots = t(tadata), raw = t(adata)),
                phenoData = AnnotatedDataFrame(pdata),
                featureData = AnnotatedDataFrame(fdata)
    )
    ## /
    
    ## Check that the new ProteomeSet is valid
    validObject(prot)
    ## /
    
    return(prot)
}
```

## 3.3 Extending MultiDataSet

So far, we have developed a function to define a new class of objects to encapsulate proteomic data. In this section, we will show how to add `ProteomeSet` objects to `MultiDataSet`. To do so, we will create a generic method for adding proteins (`add_prot`) to `MultiDataSet` and its implementation using `add_eset`.

The method `add_prot` for `MultiDataSet` will accept two arguments: a `MultiDataSet` and a `ProteomeSet`. Following S4 development rules, a new generic method for `add_prot` needs to be set:

```
setGeneric("add_prot", function(object, protSet, warnings = TRUE, ...)
    standardGeneric("add_prot")
)
```

```
## [1] "add_prot"
```

In the definition of `add_prot`, we can see the three main arguments of this function. `object` is the `MultiDataSet` where we will add the `ProteomeSet`. `protSet` is the new `ProteomeSet` that will be added. Finally, `warnings` is a flag to indicate if warnings are displayed.

The following code shows the implementation of `add_prot`. In the signature, we specify that the first argument should be a `MultiDataSet` and the second a `ProteomeSet`. If any other class is passed to the function, an error will be returned. In the code of the function, we see only two lines: a call to `add_eset` and the return of the object.

```
library(MultiDataSet)
setMethod(
    f = "add_prot",
    signature = c("MultiDataSet", "ProteomeSet"),
    definition = function(object, protSet, warnings = TRUE, ...) {
        ## Add given ProteomeSet as 'proteome'
        object <- MultiDataSet::add_eset(object, protSet, dataset.type = "proteome", GRanges = NA, ...)
        ## /
        return(object)
    }
)
```

```
## [1] "add_prot"
```

Basic method `add_eset` is used to add `eSet` derived-classes to `MultiDataSet` and accepts several arguments. Four of them are used for implementing `add_prot`. As mentioned above, the first and the second are the `MultiDataSet` object where the proteins will be added and `ProteomeSet` with the proteins data. The third, `dataset.type`, is used to tag the type of omics data that `add_prot` is adding to the `MultiDataSet`. This argument is set to `"proteome"` in `add_prot`, `"expression"` in `add_genexp` and `"snps"` in `add_snps`. The fourth argument, `GRanges` is set to `NA`. This argument can take two type of values: a `GRanges` object with the equivalent content of the `fData` included into the `ExpressionSet` or `NA` in case no genomic coordinates are available for the set’s features. In this case, since we are working with proteins, no genomic coordinates are given.

## 3.4 Data example: Adding Proteome data to MultiDataSet objects

For illustration purposes, we have created three tsv-dummy files that are used in the following code to create a `ProteomeSet` using `read_ldset` function. These files are available in the Supplementary Material of the manuscript

```
## Create a ProteomeSet with protein data
ps <- read_ldset(assayFile="assay_data.tsv", 
                 phenoFile="pheno_data.tsv",
                 featureFile="feature_data.tsv"
)
ps
```

```
## ProteomeSet (storageMode: environment)
## assayData: 5 features, 33 samples 
##   element names: prots, raw 
## protocolData: none
## phenoData
##   sampleNames: sp001 sp002 ... sp035 (33 total)
##   varLabels: gender plate kit
##   varMetadata: labelDescription
## featureData
##   featureNames: Adiponectin CRP ... APO.E (5 total)
##   fvarLabels: LoD.T LoD.B unit
##   fvarMetadata: labelDescription
## experimentData: use 'experimentData(object)'
## Annotation:
```

The created object `ps` is a `ProteomeSet` and it contains two elements called `prots` and `raw`. Moreover, `ps`’s feature data contains two columns called `LoD.B` and `LoD.T`.

Now that the proteome data is loaded and stored in a `ProteomeSet`, we can add it to a new `MultiDataSet`. `MultiDataSet` objects can be created using the constructor `createMultiDataSet`. Then the method `add_prot` is used to include the proteome data to `md`:

```
md <- createMultiDataSet()
md <- add_prot(md, ps)
```

The method `names` of `MultiDataSet` shows the datasets stored in the `MultiDataSet`. `MultiDataSet` stores datasets calling them by its data type.

```
names(md)
```

```
## [1] "proteome"
```

Finally, the *show* of the object gives more information related to the stored in the `MultiDataSet`:

```
md
```

```
## Object of class 'MultiDataSet'
##  . assayData: 1 elements
##     . elements: proteome 
##  . featureData:
##     . proteome: 5 rows, 3 cols (LoD.T, ..., LoD.B)
##  . rowRanges:
##     . proteome: NO
##  . phenoData:
##     . proteome: 33 samples, 4 cols (gender, ..., kit)
```

The name of the set is shown (`proteome`), the number of proteins (5 rows in feature data), the number of samples (33 samples in pheno data) and, since no `GRanges` was provided, `rowRanges` is `NO`.
